# University of Birmingham

**Published:** 1917-02-24

**Authors:** 


### PATRIOTISM OF MEDICAL SCHOOLS.

### University of Birmingham.

In recording examples of patriotism exhibited by the medical foundations of this country mention must be made of the public spirit displayed by the younger universities. In proportion to their size their sacrifices have been no whit less than those of the older and larger schools, while in breaking into their routine and sending out large numbers from their teaching staff they have incurred some risk of impairing a prestige but recently won. With few exceptions they have but small vested funds on which to fall back in a time when the number of students is seriously depleted. In spite of these impediments their response has been immediate and hearty to the call of the nation on their services. It may be mentioned that a School of Medicine has existed in Birmingham continuously since 1828, the school being incorporated in the University of Birmingham in 1900, when the University Charter was granted.

On the outbreak of war a large proportion of members of the medical faculty teaching staff and students (medical and dental) volunteered and were accepted for service in the Navy and Army. In addition, a considerable number of qualified past students of the school joined H.M. Forces, most of these going into the R.A.M.C. for service at home or abroad. Since then further members of the staff and students have taken up service from time to time.

Up to the present forty-eight members of the teaching staff and fifty-five medical and twenty-five dental students have joined the Services. The number of past students mobilised is not exactly known, but it is approximately 200.

It is officially known that seven students and four past students have been killed. Mr. A. J. Tonkinson, naval surgeon, went down in H.M.S. *Monmouth.* Among the past students is Miss Elizabeth Stephens Impey, M.B., Ch.B. (Birm.), who perished in the sinking of the s.s. *Persia*, which was torpedoed in the Mediterranean on December 30, 1915. Miss Impey graduated in 1911, and was on her way to India to take up the position of medical superintendent at Lady Dufferin's Women's Hospital at Lahore. It is known from survivors that to the last she was helping to save others, and that she faced death with great courage and heroism. Three students are reported missing and eight have been wounded.

The school has reason to be proud of the honours conferred on its staff and past and present students. The following may be mentioned: In October 1914 Professor J. T. J. Morrison, F.R.C.S., accompanied by Dr. Gwynne Maitland and four medical students of the University, went to Serbia as Surgeon-in-Chief of an expedition for the relief of the wounded organised by the Serbian Relief Committee, in conjunction with the St. John Ambulance Association, returning in the following spring. For his valuable services Professor Morrison was decorated by King Peter of Serbia with the Order of the White Eagle, a distinction rarely bestowed on a foreigner, and granted an honorary colonelcy in the Serbian Army. The Distinguished Service Order has been awarded to Captain E. Selby Phipson, M.B., B.S., Indian Medical Service (a past student), for conspicuous service rendered in the Dardanelles operations, and Military Crosses to the following past and present students: Captain W. Bowater, M.R.C.S., L.R.C.P., B.D.S. (R.A.M.C.), for conspicuous gallantry and devotion to duty during operations; Captain E. C. Beddows, M.R.C.S., L.R.C.P. (R.A.M.C.); Lieutenant A. C. Hincks, M.B., Ch.B. (R.A.M.C.), for conspicuous gallantry and devotion to duty at Neuve Chapelle; Lieut.-Colonel W. C. Retallack, L.D.S. (R.A.M.C.), for gallant and distinguished conduct in the field; Captain H. H. Sampson, F.R.C.S. (R.A.M.C.); Captain Pensam Thornton, M.R.C.S., L.R.C.P. (R.A.M.C.), for bravery in the field; Captain J. T. S. Weston (since killed in action), Berkshire Regiment, for conspicuous service in the field; Captain A. L. Wood, South Staffs Regiment, Special Reserve; Lieutenant M. Wykes, Royal Berkshire Regiment.

The "Medaille d'Honneur des Epidemics" has been bestowed by the French Government on Captain John Dale, M.B., Ch.B., B.Sc. (Birm.), in recognition of his work in checking an epidemic of typhoid fever amongst the civil population in France.

In addition to the foregoing, twelve students and past students have been mentioned in dispatches. It is known that there are other past and present students who have received promotion and been wounded, etc., but only those officially reported are mentioned in this statement.

